# Psychometric Validation of the Arabic Doomscrolling Scale Among Saudi Young-Adult Social Media Users: Factor Structure, Measurement Invariance Across Gender, and Digital Correlates

**DOI:** 10.3390/bs16091528

**Published:** 2026-08-30

**Authors:** Emadeldin M. Elsokkary

**Affiliations:** Department of Psychology, College of Social Sciences, Imam Mohammad Ibn Saud Islamic University (IMSIU), Riyadh 13318, Saudi Arabia; emelsokkary@imamu.edu.sa

**Keywords:** doomscrolling, Arabic validation, psychometrics, measurement invariance across gender, fear of missing out, problematic social media use, digital well-being, Saudi young-adult social media users

## Abstract

Doomscrolling has emerged as a specific pattern of digital engagement involving habitual and immersive exposure to negative online news, yet no published Arabic validation of the Doomscrolling Scale has been identified. This study aimed to translate the Doomscrolling Scale into Arabic and evaluate preliminary psychometric evidence for its use among Saudi young-adult social media users by examining its factor structure, reliability, measurement invariance across gender, and convergent, discriminant, and incremental validity. A cross-sectional online survey was conducted with 793 Saudi social media users aged 18–25 years. The sample was randomly split into exploratory factor analysis (EFA; *n* = 396) and confirmatory factor analysis (CFA; *n* = 397) subsamples. Parallel analysis and EFA supported a one-factor structure. Ordinal CFA using WLSMV in the independent CFA subsample supported the 15-item one-factor model, scaled χ^2^(90) = 216.48, *p* < 0.001, CFI = 0.976, TLI = 0.972, RMSEA = 0.060, 90% CI [0.050, 0.070], and SRMR = 0.040; standardized loadings ranged from 0.585 to 0.787. Internal consistency was high in the full sample (Cronbach’s α = 0.921), and model-based reliability was strong (McDonald’s ω/composite reliability = 0.924). Ordinal measurement invariance across gender was supported up to the strict level. Doomscrolling correlated positively with fear of missing out, problematic social media use, daily social media use, and news-checking frequency, and negatively with perceived digital well-being. In a latent incremental-validity model, doomscrolling retained a unique negative association with perceived digital well-being (β = −0.269, *p* < 0.001). The 15-item Arabic Doomscrolling Scale has preliminary support as an internally consistent and valid research measure for assessing doomscrolling among Saudi young-adult social media users.

## 1. Introduction

Digital technologies have become embedded in the everyday lives of young adults, not simply as tools for communication, but as environments in which social comparison, information seeking, emotional regulation, and identity work take place. This embeddedness makes it increasingly difficult to evaluate digital engagement in terms of screen time alone. Time spent online may support connection, learning, civic awareness, and access to social resources, yet the same connectivity may also expose users to persistent demands, emotionally charged information, and maladaptive patterns of use. Consistent with this view, reviews and large-scale analyses suggest that the relation between digital technology use and well-being is complex and depends on the form, context, and psychological function of use rather than on exposure time alone (Kushlev & Leitao, 2020; Odgers & Jensen, 2020; Orben & Przybylski, 2019). Accordingly, research has increasingly shifted from asking whether digital media use is generally “good” or “bad” to examining which forms of use, under which psychological conditions, are associated with adaptive or maladaptive outcomes.

A recent Delphi-based collective review reached a similar conclusion at the broader literature level. Capraro et al. (2025) characterized the literature on smartphone and social media use in relation to adolescent mental health as highly fragmented, with many claims limited by inconsistent evidence, non-causal designs, measurement challenges, and geographic bias. Although that review focused on adolescents rather than young adults, it underscores the need to define and measure specific forms of digital engagement rather than treating social media exposure as a homogeneous construct.

This distinction is particularly important when considering social media use. General frequency or duration of use may obscure qualitatively different patterns of engagement. Problematic social media use, for example, is usually conceptualized as a broader addiction-like pattern involving salience, mood modification, tolerance, withdrawal, conflict, and relapse (Andreassen et al., 2016). Fear of missing out (FoMO), in contrast, reflects a motivational and affective tendency to remain continuously connected because others may be having rewarding experiences from which one is absent (Przybylski et al., 2013). Digital well-being represents another distinct lens, focusing on the perceived balance between the benefits and costs of digital connectivity across emotional, social, and cognitive domains. It is not simply the absence of distress or addiction-like symptoms; rather, it concerns how users perceive digital technologies as supporting or disrupting emotional balance, social connectedness, and cognitive functioning in everyday life (Vanden Abeele, 2021; Vera Cruz et al., 2025). This makes digital well-being a particularly relevant validation criterion for doomscrolling, because repeated engagement with negative online news may be reflected in perceived emotional burden, social preoccupation, or cognitive disruption even when it is not conceptualized as clinical pathology. These constructs are related, but they do not describe the same psychological process. A person may use social media frequently without showing addiction-like symptoms, feel anxious about missing social information without compulsively consuming negative news, or report lower digital well-being because online engagement is associated with emotional, social, or cognitive disruption.

Doomscrolling has emerged as a more specific pattern within this broader ecology of digital engagement and has increasingly been treated in recent reviews as a distinct digital mental health construct rather than a loose description of excessive news consumption (Güme, 2024). Sharma et al. (2022) conceptualized doomscrolling as habitual, immersive scanning for timely negative information on social media newsfeeds. This definition separates doomscrolling from merely being informed, using social media heavily, or seeking news during uncertain events. Its defining feature is not only exposure to negative content, but also the repetitive, absorbing, and difficult-to-disengage quality of attending to such content. The construct therefore sits at the intersection of news exposure, affect regulation, online vigilance, and compulsive digital behavior. It may begin as an attempt to reduce uncertainty or stay informed, yet it can become self-reinforcing when algorithmically curated feeds repeatedly present emotionally salient, threatening, or distressing content.

A critical issue in the emerging literature is that doomscrolling should not be treated as a simple synonym for problematic social media use. Although both may involve impaired control and excessive engagement, doomscrolling is narrower in content and psychological function. It concerns persistent consumption of negative or threatening information, often in contexts of crisis, uncertainty, or social disruption. In contrast, problematic social media use can involve many types of content and motivations, including entertainment, social validation, boredom reduction, or interpersonal reassurance (Andreassen et al., 2016). This distinction has empirical implications. The original scale-development work found that doomscrolling was associated with problematic internet and social media use, online vigilance, FoMO, passive social media use, anxiety, self-control, and personality traits, but was presented as a distinct media habit rather than a redundant measure of general problematic use (Sharma et al., 2022). Subsequent studies have similarly linked doomscrolling to psychological distress, social media addiction, FoMO, life satisfaction, and mental well-being, while continuing to treat it as a separable construct (Satici et al., 2023; Soraci et al., 2025).

Preserving this conceptual distinction is important for measurement. If doomscrolling is measured only through general indicators of social media addiction, researchers may miss the specific role of negative-news engagement in digital distress and well-being. Conversely, if every form of anxious online monitoring is labeled doomscrolling, the construct risks becoming too diffuse to be useful. Recent conceptual work has therefore argued for greater precision in distinguishing doomscrolling from related practices such as doomsurfing and doomchecking. Whereas doomscrolling is typically passive, feed-based, and immersive, doomsurfing may involve more goal-directed searches for distressing information, and doomchecking may involve repeated checking of specific facts or updates (Neijzen, 2024). This distinction matters for psychometric validation because a scale should measure the intended construct with sufficient specificity, rather than blending several forms of negative information seeking into a single undifferentiated score.

The development of the Doomscrolling Scale represented a major step toward such specificity. Sharma et al. (2022) developed and refined an item pool through focus groups, expert review, exploratory factor analysis, and validation studies, resulting in a 15-item unidimensional measure and a four-item short form. The full scale was intended to capture urges to seek bad news, losing track of time while reading negative news, repeatedly refreshing feeds, continuing despite distress, and the habitual nature of negative-news browsing. Later studies provided further support for the measure in non-English contexts. In Turkish samples, Satici et al. (2023) confirmed both the 15-item and four-item versions, reported strong reliability, and showed that doomscrolling was associated with personality traits, FoMO, social media addiction, psychological distress, and indicators of well-being. In an Italian validation, Soraci et al. (2025) also supported a one-factor structure for both versions and reported theoretically consistent associations with problematic social media use, psychological distress, mental well-being, and life satisfaction. Taken together, these studies provide a clear structural expectation for the Arabic adaptation: the 15-item scale should primarily be evaluated as a unidimensional measure, while remaining open to empirical evidence of misfit or multidimensionality in the target cultural context. Although previous validation studies have also examined four-item short forms, the present study evaluates only the Arabic 15-item version. The four-item short form was not separately tested in the present sample, and no validity claim is made for an Arabic short-form score.

Cross-cultural scale adaptation requires more than literal translation. The meanings of news, crisis information, social media engagement, and emotional disclosure may vary across linguistic and cultural settings. Guidelines for test translation and adaptation emphasize the importance of maintaining conceptual, linguistic, and measurement equivalence rather than assuming that an instrument validated in one language functions identically in another (International Test Commission, 2017). For a relatively new construct such as doomscrolling, this issue is especially important. Items referring to “bad news,” “newsfeeds,” “negative news,” or feeling unable to stop scrolling may be interpreted differently depending on local media habits, platform preferences, and cultural norms regarding emotional reactions to distressing information. Therefore, an Arabic version of the Doomscrolling Scale requires empirical evaluation of its internal structure, reliability, measurement invariance, convergent validity, discriminant validity, and incremental validity.

The Arabic-speaking Saudi context provides a meaningful setting for such validation. Saudi young-adult social media users are part of a highly connected digital environment in which social media platforms are used for communication, news exposure, entertainment, and social participation. Recent national and digital-use reports indicate very high levels of internet and social media connectivity in Saudi Arabia, making the availability of culturally adapted Arabic measures particularly relevant for research on digital behavior and well-being in this context (Communications, Space and Technology Commission, 2025; Kemp, 2025). In this context, social media platforms are not only spaces for entertainment or interpersonal communication; they also serve as channels for real-time news exposure, public discussion, and rapid circulation of emotionally salient events. For Saudi young adults, exposure to negative online news may therefore occur within ordinary social media routines rather than through deliberate news seeking alone, making doomscrolling especially relevant to research on digital well-being in this population. However, the relevance of doomscrolling in this context should not be assumed from international findings alone. A validated Arabic measure is needed before researchers can estimate prevalence, examine group differences, test associations with digital well-being, or evaluate intervention needs. To the best of the author’s knowledge, no published Arabic psychometric validation of the Doomscrolling Scale was identified during targeted searches of major academic databases accessible through the Saudi Digital Library platform and other scholarly search engines conducted during manuscript preparation. The searches used combinations of the terms “doomscrolling,” “Doomscrolling Scale,” “Arabic,” “Saudi,” “validation,” “psychometric,” “factor structure,” and “measurement invariance,” supplemented by checks of reference lists from the original and available non-Arabic validation studies. This gap limits both regional research and cross-cultural comparison, particularly because the existing evidence base has been built largely from English, Turkish, Italian, and other non-Arabic samples.

The present study therefore positions psychometric validation as the central contribution, rather than treating measurement as a preliminary step before substantive modeling. Establishing the factor structure of the Arabic Doomscrolling Scale is necessary because unidimensionality is theoretically expected but cannot be assumed after translation. Reliability evidence is also required in the current sample, but internal consistency alone is insufficient. A strong validation study should also examine whether the scale operates equivalently across theoretically and practically relevant groups. In the present study, gender was selected as the grouping variable for measurement-invariance testing because gender comparisons are common in digital-behavior research and because measurement invariance is a prerequisite for interpreting such comparisons meaningfully (Putnick & Bornstein, 2016). Without invariance evidence, observed differences may reflect measurement artifacts rather than true differences in the latent construct.

External validity evidence is equally important. FoMO was selected as a theoretically relevant correlate because it captures the desire to remain continuously connected to what others are doing and to avoid missing socially relevant information (Przybylski et al., 2013). Doomscrolling may share this orientation toward ongoing monitoring, but its focus on negative or threatening information makes it conceptually narrower than FoMO. Problematic social media use, assessed through the Bergen Social Media Addiction Scale, provides another critical comparator. Because doomscrolling may resemble an addiction-like pattern of online engagement, it should correlate with problematic social media use; however, the two constructs should not be so strongly overlapping that the doomscrolling measure becomes redundant (Andreassen et al., 2016; Sharma et al., 2022). Discriminant validity relative to problematic social media use is therefore central to the present validation.

Digital well-being offers a complementary outcome-related criterion. Rather than focusing solely on distress or psychopathology, digital well-being captures the perceived emotional, social, and cognitive balance of a connected life (Vanden Abeele, 2021; Vera Cruz et al., 2025). This is particularly appropriate for doomscrolling research because the behavior may not only co-occur with anxiety or distress, but may also be associated with lower perceived control, reduced emotional balance, and diminished benefits from digital engagement. Studies have linked doomscrolling with lower mental well-being and life satisfaction, and higher psychological distress (Kaya & Griffiths, 2025; Satici et al., 2023; Soraci et al., 2025). However, these associations should be interpreted cautiously in cross-sectional research. They can support construct validity and a theoretically coherent nomological network, but they cannot establish causal direction.

From a validation perspective, the present study follows the view that validity is not established by a single statistic but by a coherent body of evidence supporting the intended interpretation and use of test scores. Accordingly, evidence from internal structure, reliability, measurement equivalence, relations with external variables, and item-level functioning was considered jointly rather than treated as independent pass/fail criteria (American Educational Research Association et al., 2014; Boateng et al., 2018).

The present study was designed to provide preliminary psychometric validation evidence for the Arabic Doomscrolling Scale among Saudi young-adult social media users. Specifically, it aimed to examine the factorial structure of the Arabic 15-item scale using exploratory and confirmatory factor analytic procedures; evaluate internal consistency and construct reliability; test measurement invariance across gender; examine convergent validity through associations with FoMO, problematic social media use, news-checking frequency, and daily social media use; evaluate discriminant validity, particularly in relation to general problematic social media use; and test incremental validity by examining whether doomscrolling shows a unique association with perceived digital well-being after accounting for theoretically related digital and demographic variables. By integrating internal structure, gender-based measurement equivalence, and external validity evidence, the study seeks to determine whether the Arabic Doomscrolling Scale shows sufficient preliminary psychometric support for research use among Saudi young-adult social media users, while recognizing the need for replication in broader Arabic-speaking populations.

## 2. Materials and Methods

### 2.1. Participants and Procedure

This study used a cross-sectional online survey design to examine the psychometric properties of the Arabic Doomscrolling Scale among Saudi young-adult social media users. Participants were recruited through social media platforms using online convenience sampling. Data were collected between January and April 2026.

Eligibility criteria required participants to be Saudi nationals, aged 18–25 years, current users of social media platforms, and willing to provide informed consent before beginning the survey. Respondents were excluded if they were outside the target age range, were non-Saudi, did not provide consent, or reported that they did not use social media. The final analytic sample comprised 793 eligible Saudi young-adult social media users. Because all survey items were set as required fields, the dataset contained no missing values for the study variables.

Before accessing the questionnaire, participants were presented with an electronic informed-consent page explaining the purpose of the study, the voluntary nature of participation, the approximate survey requirements, and their right to discontinue participation at any time. No financial or academic incentives were offered. No directly identifying information, such as names, telephone numbers, national identification numbers, or email addresses, was collected.

Ethical approval was obtained from the Institutional Review Board of Imam Mohammad Ibn Saud Islamic University (IMSIU), Saudi Arabia (Registration No. HAPO-01-R-061; Project No. 405/2025; approval date: 20 November 2025). The study protocol was approved before data collection, and all procedures were conducted in accordance with applicable institutional and national research ethics requirements.

### 2.2. Measures

#### 2.2.1. Doomscrolling Scale

Doomscrolling was assessed using the Doomscrolling Scale developed by Sharma et al. (2022). The scale consists of 15 items designed to measure habitual and immersive scanning of timely negative information on social media newsfeeds. Items are rated on a 7-point Likert-type scale ranging from 1 (strongly disagree) to 7 (strongly agree), with higher scores indicating higher levels of doomscrolling. All items are positively keyed, and no reverse-scored items are included in the final version. The present study evaluated the full 15-item version only. The four-item short form was not scored or validated in the present Arabic sample. The original scale-development study supported a unidimensional structure and reported high internal consistency (Cronbach’s α = 0.935). The scale also showed theoretically consistent associations with online vigilance, problematic internet and social media use, fear of missing out, anxiety, self-control, and relevant personality traits (Sharma et al., 2022). Further validation evidence was reported by Satici et al. (2023), who confirmed both the 15-item and 4-item versions of the scale. In their study, the 15-item version showed acceptable confirmatory factor-analytic fit (CFI = 0.92, NFI = 0.90, IFI = 0.92, SRMR = 0.046), factor loadings ranging from 0.632 to 0.835, and high reliability across three samples (Cronbach’s α = 0.938–0.944; McDonald’s ω = 0.942–0.947).

The Doomscrolling Scale was translated and culturally adapted into Arabic for the present study using a standard multistep cross-cultural adaptation procedure. First, two independent bilingual translators produced forward translations of the original English items into Arabic; one was a bilingual specialist in psychology and the other held formal qualifications in translation. The two forward translations were compared and reconciled into a single Arabic version through review of semantic, conceptual, and linguistic discrepancies. The reconciled Arabic version was then back-translated into English by an independent translator who had no access to the original English items. Minor discrepancies between the original and back-translated versions were reviewed and resolved through iterative revisions, preserving the conceptual meaning of the original items while ensuring natural Arabic wording.

The revised Arabic version was subsequently reviewed by two experts in psychology and psychometric measurement for semantic equivalence, conceptual relevance, item clarity, cultural appropriateness, and ease of understanding for Saudi young adults. This expert review served as content-validation evidence; item-level CVI statistics were not computed. Cognitive checking was then conducted with 10 Saudi participants who met the eligibility criteria for the main study, none of whom reported major comprehension problems. No items were deleted or added, and the response format was retained as in the original scale. Final changes were limited to improving the clarity and naturalness of the Arabic wording while preserving the meaning of the original items. The Arabic and English items used in the present study are provided in Section S1 of the Supplementary Material.

#### 2.2.2. Fear of Missing Out Scale

Fear of missing out was assessed using the Fear of Missing Out Scale developed by Przybylski et al. (2013). The scale consists of 10 items measuring the pervasive apprehension that others may be having rewarding experiences from which one is absent, together with the desire to remain continually connected with what others are doing. Items are rated on a 5-point Likert-type scale ranging from 1 (not at all true of me) to 5 (extremely true of me), with higher scores indicating higher levels of fear of missing out. The original validation study supported a unidimensional structure and good internal consistency (Cronbach’s α = 0.87 in Study 1 and α = 0.90 in Study 2). Construct validity was supported through associations with lower basic psychological need satisfaction, lower mood, lower life satisfaction, and higher social media engagement (Przybylski et al., 2013).

The Arabic version of the FoMO Scale was previously examined by Al-Menayes (2016) in a sample of 1327 undergraduate students. Exploratory factor analysis indicated that the data were suitable for factor analysis (KMO = 0.857; Bartlett’s test χ^2^ = 4296, df = 45, *p* < 0.001). However, unlike the original English version, the Arabic validation suggested a two-factor solution comprising eight of the ten items. The first factor included five items and explained 40.7% of the variance, whereas the second factor included three items and explained 14.6% of the variance. Internal consistency was acceptable for both factors (Cronbach’s α = 0.827 and α = 0.724, respectively), and concurrent validity was supported by positive associations with social media addiction factors (Al-Menayes, 2016).

Given this difference between the original unidimensional structure and the available Arabic validation evidence, the factor structure and reliability of the FoMO Scale were re-examined in the present sample before using it as a convergent-validity criterion. In the current data, parallel analysis retained a single factor, and standardized loadings were positive and statistically significant, ranging from 0.533 to 0.689. Internal consistency was good (Cronbach’s α = 0.860), and model-based reliability was comparable (CR/ω = 0.861; AVE = 0.385). Accordingly, the total FoMO score was used as an external validity criterion and as a latent predictor in the incremental-validity model.

#### 2.2.3. Bergen Social Media Addiction Scale

Problematic social media use was assessed using the Bergen Social Media Addiction Scale (BSMAS), originally adapted from the Bergen Facebook Addiction Scale and generalized to social media use by Andreassen et al. (2016). The scale consists of six items reflecting the core components of behavioral addiction: salience, tolerance, mood modification, relapse, withdrawal, and conflict. Items are rated on a 5-point Likert-type scale ranging from 1 (very rarely) to 5 (very often), referring to experiences during the past year. Higher scores indicate higher levels of addictive or problematic social media use. In Andreassen et al. (2016), the social networking version demonstrated good internal consistency (Cronbach’s α = 0.88) in a large adult sample.

The Arabic version of the BSMAS was validated by El Abiddine et al. (2025) in a sample of 757 Algerian university students. Confirmatory factor analysis supported a unidimensional structure (CFI = 0.966, TLI = 0.943), and Rasch analysis supported adequate item functioning, with infit mean-square values ranging from 0.83 to 1.16 and outfit mean-square values ranging from 0.82 to 1.15. Internal consistency was acceptable (Cronbach’s α = 0.74; McDonald’s ω = 0.78), factor loadings ranged from 0.402 to 0.670, and corrected item–total correlations ranged from 0.349 to 0.529. The Arabic BSMAS also demonstrated measurement invariance across genders. Criterion-related validity was supported by positive associations with depressive symptoms measured by the CES-D (r = 0.290, *p* < 0.001) and negative associations with life satisfaction measured by the SWLS (r = −0.232, *p* < 0.001).

In the present study, the BSMAS was included to examine the convergent and discriminant validity of the Arabic Doomscrolling Scale, particularly to determine whether doomscrolling is empirically distinguishable from general problematic social media use. In the current sample, the BSMAS showed acceptable internal consistency (Cronbach’s α = 0.744). In the multi-construct maximum likelihood measurement models used for discriminant-validity and incremental-validity analyses, standardized loadings ranged from 0.475 to 0.685, with CR/ω = 0.750 and AVE = 0.337. These values were considered sufficient for using the BSMAS as a comparison construct, while the relatively low AVE was taken into account when interpreting Fornell–Larcker evidence.

#### 2.2.4. Arabic Perceived Digital Well-Being Scale

Perceived digital well-being was assessed using the Arabic version of the Perceived Digital Well-Being Scale (PDWS). The PDWS is based on the Perceived Digital Well-Being in Adolescence Scale developed by Rosič et al. (2024) and later adapted and validated for young adults by Vera Cruz et al. (2025). The scale consists of 17 bipolar items assessing perceived smartphone-related digital well-being across three domains: emotional digital well-being, social digital well-being, and cognitive digital well-being. Each item is rated on a 5-point bipolar response scale, with lower scores reflecting negative perceived consequences of smartphone use and higher scores reflecting positive perceived consequences of smartphone use. Higher domain and total scores indicate higher perceived digital well-being.

The English young-adult version of the PDWS showed good psychometric properties in samples from the United States and the United Kingdom. Confirmatory factor analysis supported the three-factor structure, internal consistency was satisfactory across the three domains, convergent validity was supported through associations with digital flourishing and digital stress, and measurement invariance was supported across national samples, although strict invariance across gender was not fully supported (Vera Cruz et al., 2025).

The Arabic PDWS used in the present study has been psychometrically validated in a Saudi young-adult sample by Elsokkary (2026). In that validation study, a cross-sectional instrument-validation design was used with 450 Saudi young adults aged 18–25 years. The full sample was randomly split into two independent subsamples: Sample 1 for exploratory factor analysis (*n* = 225) and Sample 2 for confirmatory factor analysis (*n* = 225). Exploratory factor analysis supported a three-factor structure, and confirmatory factor analysis supported a well-fitting second-order model: χ^2^(116) = 133.275, *p* = 0.130, χ^2^/df = 1.149, CFI = 0.986, TLI = 0.983, RMSEA = 0.026, and SRMR = 0.055. Convergent validity was supported by positive associations with life satisfaction and negative associations with fear of missing out across both subsamples. Internal consistency was acceptable to strong across domains and total scores, with Cronbach’s alpha ranging from 0.759 to 0.886 and McDonald’s omega ranging from 0.761 to 0.889. Measurement invariance was supported across gender and education level.

In the present study, the Arabic PDWS was included as a theoretically proximal indicator of perceived digital well-being, given that doomscrolling reflects a maladaptive pattern of negative news consumption within digital and social media environments. Because the Arabic PDWS was validated in a recently published study by the present author, it was treated as a domain-proximal validity criterion rather than as a substitute for more established Arabic measures of psychological distress, life satisfaction, anxiety, depression, sleep, or digital stress. Internal consistency in the current sample was satisfactory for the total score (Cronbach’s α = 0.887) and for the emotional (α = 0.860), social (α = 0.801), and cognitive (α = 0.764) domains. In the incremental-validity model, perceived digital well-being was represented as a latent variable using the three domain scores as indicators; the standardized domain loadings ranged from 0.674 to 0.709, with CR/ω = 0.729 and AVE = 0.473.

### 2.3. Statistical Analysis

Data screening first verified response-range validity, missingness, and univariate item distributions. Absolute skewness and kurtosis were inspected to evaluate whether item distributions showed severe departures from normality before applying normal-theory maximum likelihood estimation. Additional data-quality checks examined duplicate participant IDs, fully duplicated response vectors across all 48 item responses, invariant responding across all items, within-person response variability, longstring indices, within-scale straight-lining, and perfect alternating response patterns. Completion time, IP address, device, and session metadata were not retained; therefore, implausibly short completion-time checks and direct automated bot-screening procedures could not be conducted.

For cross-validation, the analytic sample was randomly partitioned using a fixed seed (12345) into an exploratory factor analysis (EFA) subsample and an independent confirmatory factor analysis (CFA) subsample. Equivalence between the two subsamples was examined across key demographic and behavioral variables before proceeding with the factor-analytic analyses.

In the EFA subsample, factorability was evaluated using the Kaiser–Meyer–Olkin index and Bartlett’s test of sphericity. The number of factors was determined using Horn’s parallel analysis with 1000 simulated correlation matrices. Maximum likelihood extraction was used for the one-factor exploratory solution, and a two-factor oblique solution was examined as a sensitivity check for possible multidimensionality.

Because the items are ordered categorical, the one-factor structure was tested in the independent CFA subsample using the robust weighted least squares mean- and variance-adjusted estimator (WLSMV), with DS1–DS15 specified as ordered-categorical indicators. In lavaan, WLSMV is implemented as diagonally weighted least squares with a scaled-and-shifted mean- and variance-adjusted test statistic. Maximum likelihood (ML) estimation was retained only as convergent evidence to evaluate whether the same one-factor structure and loading pattern were obtained under a continuous-indicator estimator.

Internal consistency was evaluated using Cronbach’s alpha in the full sample and in the CFA subsample. Model-based reliability was evaluated using McDonald’s ω and composite reliability (CR). Average variance extracted (AVE) was interpreted cautiously when it was marginally below the conventional 0.50 guideline, with reference to the full pattern of evidence, including CR, standardized loadings, and theoretically expected external associations (cf. Fornell & Larcker, 1981).

Measurement invariance was examined across gender only in the ordinal CFA framework using WLSMV, theta parameterization, and the Wu and Estabrook (2016) identification approach for ordered-categorical indicators. The sequence tested configural invariance, equal thresholds, equal thresholds and loadings, and strict invariance with equal residual variances. Invariance across age and education was not tested because of the restricted age range and unequal subgroup sizes; therefore, no claim of invariance across age or education was made. Model comparisons were evaluated using changes in approximate fit indices, particularly ΔCFI, ΔRMSEA, and ΔSRMR, following commonly used recommendations for measurement-invariance evaluation (Cheung & Rensvold, 2002; Chen, 2007), together with scaled-and-shifted chi-square difference tests using the Satorra and Bentler (2010) correction.

Before being used as validity criteria, the external measures were examined for psychometric adequacy in the current sample. Convergent and criterion-related validity were evaluated using correlations between doomscrolling and theoretically relevant external criteria. Pearson correlations were used for continuous variables, whereas Spearman’s rho was used for the ordinal news-checking frequency variable.

Discriminant validity was evaluated using latent correlations and the heterotrait–monotrait ratio (HTMT), with the Fornell–Larcker criterion treated as supportive rather than decisive evidence. For the key comparison with problematic social media use, an additional ordinal WLSMV discriminant-validity test was conducted in the full sample (*N* = 793), with items specified as ordered-categorical indicators. This test compared a two-factor model in which the Doomscrolling Scale and BSMAS factors were freely correlated with a nested model in which their latent correlation was fixed to 1.0. A significant scaled-and-shifted chi-square difference test and deterioration in approximate fit were interpreted as evidence that the two constructs were empirically distinguishable, while still allowing for substantial conceptual and empirical overlap. Incremental validity was tested using a latent structural model in which perceived digital well-being was represented by its three domain scores and regressed on latent doomscrolling, fear of missing out, and problematic social media use, together with observed demographic and behavioral covariates. The observed covariates were coded as follows. Gender was coded 1 = male and 2 = female, so the gender coefficient reflects the direction of higher values for female participants. Education level was coded ordinally as 1 = high school or less, 2 = undergraduate/bachelor, and 3 = postgraduate. News-checking frequency was coded ordinally as 1 = rarely, 2 = sometimes, 3 = often, and 4 = very often. Age and daily social media use were entered as continuous observed covariates. Because all substantive variables were self-reported in a single cross-sectional survey, the incremental-validity model was used only to evaluate whether doomscrolling showed a unique association with perceived digital well-being after adjustment for related constructs and covariates. The model was not interpreted as evidence of temporal prediction, causal influence, or absence of shared-method variance.

Supplementary item-level evidence was obtained using a graded response model (GRM) fitted to the 15 doomscrolling items in the full sample. Differential item functioning (DIF) across gender was examined using ordinal logistic regression. These analyses were treated as supplementary evidence complementing the factor-analytic and measurement-invariance findings, not as substitutes for them.

The study was not preregistered. Analyses were therefore described according to their role in the validation design rather than as preregistered hypotheses. The primary confirmatory analyses were the one-factor ordinal CFA of the 15-item Arabic Doomscrolling Scale, ordinal measurement invariance across gender, reliability estimation, and validity analyses based on theoretically expected associations with fear of missing out, problematic social media use, news-checking frequency, daily social media use, and perceived digital well-being. The EFA, parallel analysis, two-factor sensitivity solution, GRM, DIF analyses, additional WLSMV discriminant-validity comparison, and data-quality checks were treated as exploratory, sensitivity, or supplementary analyses intended to evaluate robustness, response quality, and item-level functioning.

## 3. Results

### 3.1. Participant Characteristics and Data Screening

The analytic sample comprised 793 Saudi young-adult social media users aged 18–25 years. All item responses were within the valid response ranges, and no missing values were present. Additional data-quality checks were conducted using the available item-response dataset. No duplicate participant IDs or fully duplicated 48-item response vectors were detected. No respondent gave the same response to all 48 items, and the minimum within-person standard deviation across the 48 items was 0.866. No perfect alternating response patterns were observed. The overall longstring index had a median of 4 and a maximum of 15. Within-scale straight-lining was rare: one participant on the Doomscrolling Scale (0.1%), one participant on the FoMO Scale (0.1%), seven participants on the BSMAS (0.9%), and no participants on the PDWS. These patterns were judged to be limited and plausible, and no cases were excluded on this basis. Item distributions showed no severe departures from normality; absolute skewness values ranged from 0.12 to 0.29, and absolute kurtosis values ranged from 0.10 to 0.78. As shown in Table 1, the sample was nearly balanced by gender and was composed primarily of undergraduate or bachelor-level participants. Mean age was 21.83 years (SD = 1.92), and mean daily social media use was 4.94 h (SD = 1.70, range = 1.6–12.0). On the composite measures, the mean doomscrolling score was 4.36 (SD = 1.06) on the 7-point response metric. Mean scores on the 5-point measures were 3.08 (SD = 0.78) for fear of missing out, 2.93 (SD = 0.75) for problematic social media use, and 3.15 (SD = 0.72) for perceived digital well-being.

### 3.2. Factor Structure and Reliability of the Arabic Doomscrolling Scale

The sample was randomly divided using a fixed seed (12345) into an exploratory factor analysis subsample (*n* = 396) and an independent confirmatory factor analysis subsample (*n* = 397). The two subsamples did not differ significantly on key demographic or behavioral variables, supporting their use for cross-validation (Supplementary Table S3).

In the EFA subsample, the items were well suited to factor analysis (KMO = 0.957; Bartlett χ^2^(105) = 2600.90, *p* < 0.001). Horn’s parallel analysis retained a single factor: the first observed eigenvalue (7.07) exceeded its 95th-percentile random counterpart (1.41), whereas the second observed eigenvalue (1.01) fell below the corresponding random value (1.32). The first factor accounted for 47.1% of the total item variance, and the one-factor maximum likelihood solution explained 43.5% of the common variance. All EFA loadings were salient (range = 0.480–0.747). A two-factor oblique sensitivity solution yielded a high factor correlation (r = 0.82) and did not produce a substantively interpretable simple structure, further supporting essential unidimensionality.

**Figure 1:**
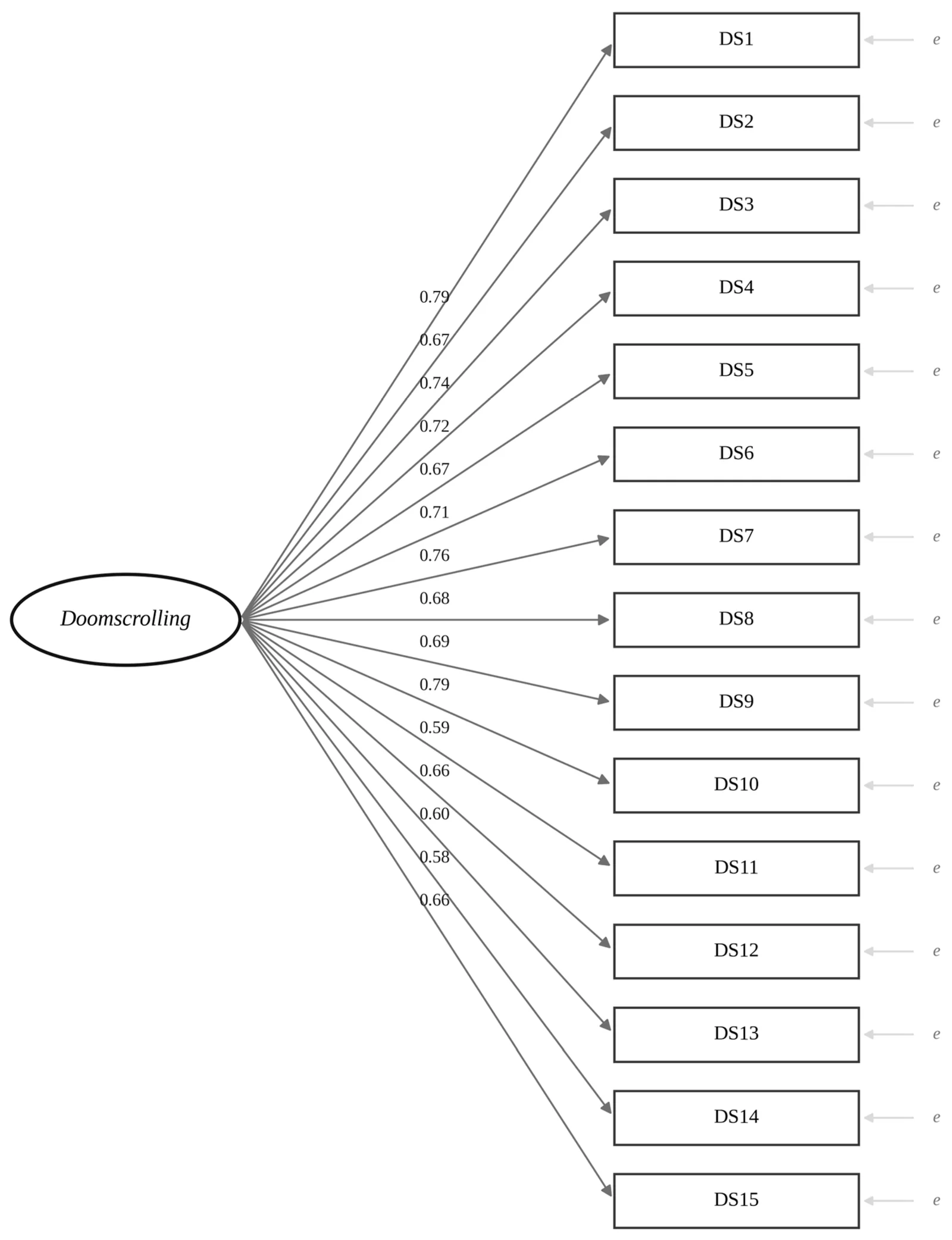
One-Factor Confirmatory Model of the Arabic Doomscrolling Scale. Note. Values are standardized factor loadings from the primary ordinal WLSMV confirmatory model in the CFA subsample (*n* = 397). DS1–DS15 represent the 15 Doomscrolling Scale items. Error terms are shown for completeness. No correlated residuals were specified.

The one-factor solution was then tested in the independent CFA subsample using WLSMV, with the 15 items specified as ordered-categorical indicators. The ordinal WLSMV model fit the data well: scaled χ^2^(90) = 216.48, *p* < 0.001, CFI = 0.976, TLI = 0.972, RMSEA = 0.060, 90% CI [0.050, 0.070], SRMR = 0.040. The robust indices were consistent with this conclusion (CFI = 0.959, TLI = 0.952, RMSEA = 0.059, 90% CI [0.047, 0.070]). All standardized loadings were statistically significant (*p* < 0.001) and ranged from 0.585 to 0.787 (Supplementary Table S2). The standardized one-factor confirmatory model is displayed in Figure 1. The supplementary ML solution converged on the same one-factor structure (CFI = 0.959, RMSEA = 0.055, SRMR = 0.081, loading range = 0.574–0.767), but because SRMR was marginally above the conventional 0.080 guideline and the indicators were ordered categorical, the ML solution is reported only as convergent evidence rather than as the primary estimator. Local diagnostics did not indicate substantively meaningful localized misfit; therefore, no correlated residuals or post hoc model modifications were introduced.

Internal consistency and model-based reliability were high. Cronbach’s alpha was 0.921 in the full sample and 0.923 in the CFA subsample, and McDonald’s ω/composite reliability (CR) was 0.924. The WLSMV-based AVE was 0.476 and the ML-based AVE was 0.449. Because AVE was marginally below the conventional 0.50 guideline under both estimators, convergent validity was interpreted cautiously based on the full pattern of evidence. The marginal shortfall in AVE is not in itself decisive against convergent validity, given the high composite reliability (CR = 0.924), the uniformly salient and significant standardized loadings, and the theoretically consistent pattern of external associations (cf. Fornell & Larcker, 1981).

Table 2 summarizes the factor-analytic and reliability evidence for the Arabic Doomscrolling Scale.

### 3.3. Measurement Invariance Across Gender

Measurement invariance was examined across gender, the grouping variable in this validation study, using the ordinal WLSMV framework in the CFA subsample (male *n* = 205, female *n* = 192). Invariance across age and education was not tested, given the restricted age range (18–25 years) and unequal subgroup sizes; therefore, no claim of invariance across these variables is made. Following the Wu and Estabrook (2016) identification sequence for ordered-categorical indicators, the models tested configural invariance, equal thresholds, equal thresholds and loadings, and strict invariance with equal residual variances. As shown in Table 3, fit changes across increasingly constrained models were negligible (all ΔCFI ≤ 0.006, |ΔRMSEA| ≤ 0.009, and ΔSRMR ≤ 0.003), and all scaled-and-shifted chi-square difference tests were non-significant. These findings support ordinal measurement invariance up to the strict level, indicating that thresholds, loadings, and residual variances operated equivalently for male and female participants in the present sample.

### 3.4. Convergent, Criterion-Related, and Discriminant Validity

The external measures showed acceptable psychometric adequacy in the present sample before being used as validity criteria, including documented internal consistency, loading ranges, CR/ω, and AVE where relevant (Supplementary Table S4). In the full sample, doomscrolling correlated positively with fear of missing out, problematic social media use, daily social media hours, and news-checking frequency, and negatively with perceived digital well-being (Table 4, Panel A). These associations were statistically significant and in theoretically expected directions. Because news-checking frequency was ordinal, its association with doomscrolling is reported as Spearman’s rho; a Spearman sensitivity estimate for the doomscrolling-hours association was consistent (ρ = 0.237), indicating that the Pearson estimate was not driven by distributional irregularity.

Discriminant validity was evaluated using latent correlations, HTMT, the Fornell–Larcker criterion as supportive evidence, and an additional ordinal WLSMV nested-model comparison for the key comparison with problematic social media use (Table 4, Panels B and C). Doomscrolling and problematic social media use were substantially related at the latent level (r = 0.624, r^2^ = 0.389), and the Fornell–Larcker criterion was not satisfied for problematic social media use because of the comparatively low AVE of that measure. However, HTMT was below the conservative 0.85 threshold (HTMT = 0.627), indicating that the constructs were not redundant. The additional ordinal WLSMV comparison in the full sample (*n* = 793) provided stronger evidence of empirical distinguishability: the freely correlated two-factor model fit the data well, whereas fixing the doomscrolling–problematic-social-media-use latent correlation to 1.0 produced substantially worse fit; the scaled-and-shifted chi-square difference test was significant, Δχ^2^(1) = 78.04, *p* < 0.001. The WLSMV latent correlation was substantial (r = 0.626, 95% CI [0.571, 0.682]) but clearly below 1.0. Thus, the two constructs should be interpreted as strongly related but empirically distinguishable, rather than independent. For the doomscrolling–fear-of-missing-out comparison, all indices supported discriminant validity (latent r = 0.433, r^2^ = 0.187, HTMT = 0.438).

### 3.5. Incremental Validity in Relation to Perceived Digital Well-Being

Incremental validity was tested in the full sample using a latent structural model in which perceived digital well-being was specified as a latent variable represented by its three domain scores. Perceived digital well-being was regressed on latent doomscrolling, fear of missing out, and problematic social media use, together with observed covariates (daily social media hours, news-checking frequency, gender, age, and education). The model fit the data well, χ^2^(701) = 1046.69, χ^2^/df = 1.49, CFI = 0.964, TLI = 0.962, RMSEA = 0.025, and accounted for 29.2% of the variance in perceived digital well-being.

As shown in Table 5, doomscrolling retained a significant unique negative association with perceived digital well-being after adjustment for the other constructs and covariates (β = −0.269, *p* < 0.001). This association was comparable in magnitude to the unique association for problematic social media use (β = −0.277, *p* < 0.001), whereas fear of missing out was not statistically significant after adjustment (β = −0.075, *p* = 0.163). None of the observed covariates were significantly associated with perceived digital well-being in the adjusted model. Because all variables were self-reported and the design was cross-sectional, these findings are interpreted as evidence of incremental validity and unique association only. They do not establish temporal prediction or causal effects, and shared-method variance cannot be ruled out.

### 3.6. Supplementary Item Response Theory and Differential Item Functioning Evidence

As supplementary evidence, a graded response model was fitted to the 15 doomscrolling items in the full sample. Item discrimination parameters were all greater than 1.00 (range = 1.13–2.09, M = 1.62), and ordered thresholds covered a broad range of the latent trait (approximately −3.8 to +2.9). Gender DIF was examined using ordinal logistic regression. DIF was negligible for all items: the largest ΔR^2^ was 0.002, no item exceeded the Jodoin–Gierl negligible-DIF threshold, and no item remained statistically significant after Bonferroni correction (Supplementary Table S5). These findings complement, but do not replace, the latent measurement-invariance results reported above.

## 4. Discussion

The present study provides preliminary psychometric validation evidence for the Arabic Doomscrolling Scale among Saudi young-adult social media users. Overall, the findings provide initial support for the 15-item Arabic version as a coherent, essentially unidimensional, internally consistent, and valid research measure of habitual engagement with negative online news in this population. The evidence was consistent across several complementary levels of evaluation: internal structure, reliability, gender-based measurement invariance, convergent and discriminant validity, incremental validity, and supplementary item-level analyses. Importantly, the results support the interpretation of doomscrolling as a specific digital behavior that is related to, but not reducible to, fear of missing out or general problematic social media use.

The factor-analytic findings provide generally supportive evidence for the unidimensional structure of the Arabic Doomscrolling Scale. Parallel analysis retained a single factor, the exploratory factor analysis showed salient loadings across all items, and the independent ordinal WLSMV confirmatory factor analysis supported the one-factor model without requiring correlated residuals or post hoc modifications. This support should nevertheless be interpreted alongside the marginal AVE shortfall and the supplementary ML SRMR value, rather than as uniformly strong evidence across all indices. This pattern is consistent with the original development of the Doomscrolling Scale by Sharma et al. (2022), in which doomscrolling was conceptualized as a coherent media habit involving habitual and immersive scanning for negative information on social media newsfeeds. It also aligns with later validation studies in Turkish and Italian samples, which supported the one-factor structure of the 15-item version and showed theoretically meaningful associations with social media addiction, fear of missing out, psychological distress, and well-being-related indicators (Satici et al., 2023; Soraci et al., 2025). The absence of a substantively interpretable two-factor solution in the present study further suggests that the translated items reflect a common underlying construct rather than separable dimensions of negative-news engagement. This is important from a cross-cultural adaptation perspective, because it indicates that the Arabic translation preserved the intended conceptual structure of the scale rather than introducing linguistic or cultural fragmentation across item content, consistent with principles of test adaptation that emphasize conceptual and measurement equivalence across languages (International Test Commission, 2017).

The reliability evidence was strong with respect to internal consistency and model-based reliability. Internal consistency was high in both the full sample and the confirmatory subsample, and model-based reliability was similarly strong; however, the present study did not assess test–retest reliability, so the temporal stability of Arabic Doomscrolling Scale scores remains unknown. These findings indicate that the Arabic items function as a homogeneous item set and provide internally consistent measurement of the latent construct. At the same time, the interpretation of convergent evidence from the measurement model should remain appropriately cautious because the average variance extracted remains marginally below the conventional 0.50 guideline. This shortfall does not, in itself, invalidate the measure, particularly given the high composite reliability, the uniformly salient and statistically significant standardized loadings, and the theoretically consistent pattern of external associations. This interpretation is consistent with Fornell and Larcker’s (1981) view that AVE should be considered alongside composite reliability and the broader validity evidence rather than treated as an isolated pass/fail criterion. Accordingly, the present evidence supports the internal coherence of the Arabic Doomscrolling Scale while acknowledging that additional validation in independent Arabic-speaking samples would further strengthen the evidence for convergent validity.

Measurement invariance analyses provided additional support for the use of the Arabic Doomscrolling Scale across gender. The scale demonstrated invariance up to the strict level, indicating that the same latent construct was measured in a comparable way among male and female participants in the present sample. This finding is important because measurement invariance is a prerequisite for meaningful group comparisons; without such evidence, observed differences may reflect measurement artifacts rather than true differences in the latent construct (Putnick & Bornstein, 2016). The present results therefore suggest that latent gender comparisons would be psychometrically permissible in similar samples of Saudi young-adult social media users. However, the scope of this evidence should not be overstated. Measurement invariance was examined across gender only; invariance across age and education was not tested because of the restricted age range and unequal subgroup sizes. Thus, the present findings support gender-based measurement equivalence but do not establish equivalence across other demographic groups.

The pattern of external associations supports the nomological validity of the Arabic Doomscrolling Scale. Doomscrolling was positively associated with fear of missing out, problematic social media use, daily social media use, and news-checking frequency, and negatively associated with perceived digital well-being. These findings are theoretically coherent. FoMO reflects a motivational tendency to remain connected to ongoing social information and may therefore overlap with the monitoring component of doomscrolling, even though doomscrolling is more specifically oriented toward negative or threatening information (Przybylski et al., 2013; Sharma et al., 2022). Similarly, problematic social media use reflects a broader addiction-like pattern involving salience, tolerance, withdrawal, conflict, and impaired control (Andreassen et al., 2016). Doomscrolling may share some of these behavioral features, such as difficulty disengaging or repeated checking, while remaining narrower in content and affective function. The positive association with news-checking frequency also supports the interpretation of doomscrolling as linked to repeated engagement with current information, whereas the negative association with perceived digital well-being is consistent with the idea that habitual negative-news exposure may be embedded in less favorable emotional, social, and cognitive experiences of digital life (Vanden Abeele, 2021; Vera Cruz et al., 2025).

This pattern also extends previous validation findings by showing that the expected nomological network of doomscrolling can be observed in an Arabic-speaking Saudi young-adult sample. Consistent with Turkish and Italian validation studies (Satici et al., 2023; Soraci et al., 2025), doomscrolling was related to adjacent digital constructs such as fear of missing out and problematic social media use, while also showing a negative association with well-being-related indicators. The association with news-checking frequency is particularly important because it supports the content-specific nature of the construct: doomscrolling is not merely frequent social media use, but a pattern of repeated engagement with negative or threatening online information. In the present Saudi context, this finding suggests that doomscrolling may be embedded in everyday social media routines through which young adults monitor, encounter, and remain engaged with negative news content.

The evidence for discriminant validity relative to general problematic social media use should be interpreted cautiously but positively. Doomscrolling and problematic social media use were substantially related, which is theoretically expected because both involve maladaptive patterns of social media engagement and difficulty disengaging. Moreover, the Fornell–Larcker comparison was not satisfied on the problematic-social-media-use side, partly reflecting the comparatively low AVE of the BSMAS in the current measurement model. However, several pieces of evidence indicate that the two constructs were not redundant. HTMT remained below the conservative threshold, the ordinal WLSMV nested comparison showed that a freely correlated two-factor model fit substantially better than a model fixing the latent correlation to 1.0, and the latent correlation, although substantial, was clearly below unity. In addition, the incremental-validity model showed that doomscrolling retained a unique negative association with perceived digital well-being after adjustment for problematic social media use and other covariates. Taken together, these findings support a cautious interpretation: doomscrolling appears to be strongly related to, but empirically distinguishable from, general problematic social media use. This distinction is theoretically meaningful because doomscrolling captures repetitive engagement with negative online news, whereas problematic social media use reflects broader addiction-like engagement with social media platforms (Andreassen et al., 2016; Sharma et al., 2022).

The incremental-validity model is consistent with this cautious interpretation. Doomscrolling retained a unique negative association with perceived digital well-being after accounting for fear of missing out, problematic social media use, daily social media use, news-checking frequency, gender, age, and education. The magnitude of this unique association was comparable to that of problematic social media use, whereas fear of missing out was no longer statistically significant in the adjusted model. Within the limits of a cross-sectional self-report validity model, this pattern suggests that doomscrolling contributes distinctive validity-relevant information about perceived digital well-being beyond adjacent digital constructs. This evidence should be understood as domain-proximal, however, rather than as external validation against established mental-health, sleep, or life-satisfaction outcomes. In other words, the specific habit of repeatedly engaging with negative online news appears to capture a form of digital experience that is not fully represented by general social media addiction-like symptoms or by the desire to remain socially connected. This interpretation is consistent with research linking doomscrolling to lower well-being and higher psychological distress (Kaya & Griffiths, 2025; Satici et al., 2023; Soraci et al., 2025). However, because the present study used a cross-sectional design, the findings should be interpreted strictly as unique associations within a validity model. They do not establish whether doomscrolling leads to lower digital well-being, whether lower digital well-being increases vulnerability to doomscrolling, or whether both are shaped by other psychological or contextual factors.

This cautious interpretation is consistent with recent synthetic work on digital media and mental health. Capraro et al. (2025) used a Delphi process to synthesize expert judgments on commonly cited claims about smartphone and social media use and adolescent mental health, concluding that the broader evidence base remains fragmented and is shaped by measurement, causal-inference, and geographic limitations. The present study addresses one part of this broader challenge by focusing on the measurement of a specific digital behavior—habitual engagement with negative online news—rather than treating social media use as a single undifferentiated exposure. At the same time, the findings should not be read as resolving broader causal debates about digital media and mental health.

The supplementary item-level analyses provide additional support for the scale’s psychometric functioning. The graded response model indicated adequate item discrimination across all items, suggesting that the items differentiated respondents across levels of the latent trait. The absence of meaningful gender differential item functioning complements the multigroup measurement-invariance findings by showing that individual items did not show practically important gender-related bias. These results strengthen confidence that the Arabic items function fairly across male and female respondents in the present sample. Nevertheless, these analyses should be understood as supplementary evidence. They support, but do not replace, the primary factor-analytic and measurement-invariance results.

The study has several theoretical and practical implications. Theoretically, it contributes to the refinement of doomscrolling as a distinct construct within the broader ecology of digital engagement. The results support the view that doomscrolling is neither reducible to time spent online nor interchangeable with general problematic social media use. Rather, it appears to represent a specific pattern of repetitive engagement with negative online information. This distinction is important for future research on digital well-being, because different digital behaviors may have different psychological correlates and implications. Practically, this preliminary Arabic validation enables researchers to examine doomscrolling with greater precision among Saudi young-adult social media users, but further validation is needed before extending score interpretations to broader Arabic-speaking populations. It may support studies estimating the prevalence of doomscrolling, testing group differences where measurement invariance is established, examining associations with mental health and digital well-being outcomes, and evaluating educational or preventive interventions targeting maladaptive news-consumption habits. The scale should be used as a research instrument rather than as a clinical diagnostic tool, because the present evidence does not establish clinical cutoffs or diagnostic validity.

Several limitations should be considered. First, the study used a cross-sectional design. Therefore, the associations observed in the validity and incremental-validity analyses cannot establish causal direction, temporal ordering, or predictive validity over time. Future longitudinal studies are needed to examine whether doomscrolling predicts later changes in digital well-being, distress, or other psychological outcomes. This cross-sectional limitation is also relevant to reliability evidence, because the study did not include a test–retest design. Therefore, the reliability evidence is limited to internal consistency and model-based reliability, and the temporal stability of Arabic Doomscrolling Scale scores remains to be established. Second, although the sample was large, it was obtained through online convenience sampling and was not probability-based or nationally representative. The sample was also restricted to Saudi young-adult social media users aged 18–25 years. This sampling frame is appropriate for the present preliminary validation purpose but limits generalizability to older adults, adolescents, clinical populations, non-Saudi Arabic-speaking groups, broader Arabic-speaking populations, and individuals with limited or no social media use. Accordingly, the findings should not be interpreted as population-level norms, prevalence estimates, or evidence that applies to all Saudi or Arabic-speaking adults. Third, all substantive measures were based on self-report within the same online survey. This may introduce shared-method variance, recall bias, or social desirability effects, and it is particularly relevant to the incremental-validity model, in which both predictors and perceived digital well-being were reported by the same respondents at the same time. In addition, because response-time, IP-address, device, and session metadata were not retained, the study could not directly evaluate implausibly short completion times or automated bot responding. Future studies could incorporate behavioral indicators, ecological momentary assessment, digital trace data, informant-based measures, and more extensive response-quality metadata where ethically and practically feasible. Fourth, measurement invariance was tested only across gender. Further work should examine invariance across age groups, education levels, countries, dialect communities, and other culturally meaningful subgroups. Fifth, although the primary ordinal WLSMV CFA supported the one-factor structure and showed acceptable-to-good approximate fit, the measurement evidence should still be interpreted conservatively because AVE was marginally below 0.50 and the supplementary ML solution had a marginal SRMR value (0.081). These findings should be replicated in independent Arabic-speaking samples and, where appropriate, compared across alternative categorical estimation frameworks. Sixth, the external validity evidence relied in part on the Arabic PDWS as a domain-proximal indicator of perceived digital well-being. Although this measure was theoretically appropriate for the present validation purpose and showed satisfactory reliability in the current sample, its Arabic validation was recently published by the present author. The study also did not include more established Arabic measures of psychological distress, life satisfaction, anxiety, depression, sleep quality, or digital stress. Consequently, the incremental-validity findings should be interpreted as evidence that doomscrolling is uniquely associated with perceived digital well-being in this sample, not as evidence of broader criterion validity across established mental-health or behavioral outcomes. The study also did not include clinical diagnostic criteria or external behavioral benchmarks; therefore, no clinical cutoff scores or diagnostic interpretations should be inferred from the present findings.

Future research should build on the present validation in several directions. Longitudinal studies are needed to evaluate temporal stability, test–retest reliability, and prospective associations with digital well-being using both domain-specific digital well-being measures and more established Arabic measures of life satisfaction, psychological distress, anxiety, depressive symptoms, sleep quality, digital stress, and other health-related outcomes. Cross-cultural studies across Arabic-speaking countries would help determine whether the scale functions similarly across dialects, media environments, and sociocultural contexts. Future work should also test measurement invariance across broader age ranges, educational groups, and platform-use profiles. In addition, studies using experience-sampling methods could clarify when and why individuals engage in doomscrolling episodes and how such episodes relate to momentary affect and perceived control over digital behavior. Further research may also examine whether doomscrolling is empirically distinguishable from related practices such as doomsurfing and doomchecking, as recent conceptual work suggests that these behaviors may differ in intentionality, content, and mode of information seeking (Neijzen, 2024). Finally, future studies should evaluate the Arabic four-item short form separately before it is used in Arabic-speaking samples, particularly for large-scale surveys where respondent burden is a concern. The present findings support only the 15-item Arabic version and should not be taken as validation evidence for a short-form score.

## 5. Conclusions

The present study provides preliminary evidence that the 15-item Arabic Doomscrolling Scale is a psychometrically sound research measure for assessing doomscrolling among Saudi young-adult social media users. The scale showed an essentially unidimensional structure, strong internal consistency, strong model-based reliability, measurement invariance across gender, theoretically coherent external associations, discriminant validity relative to general problematic social media use, and incremental validity in relation to perceived digital well-being. Supplementary item-level analyses further supported adequate item functioning and negligible gender DIF. These findings address an important gap in the Arabic digital-behavior literature by providing preliminary evidence for a culturally adapted measure of doomscrolling for research use in the present Saudi young-adult context. At the same time, the scale should be applied with appropriate caution: the present evidence is cross-sectional, based on self-report data, and limited to Saudi young-adult social media users. Additional longitudinal, cross-cultural, and criterion-validity studies are needed to extend the evidence base beyond Saudi young-adult social media users and clarify the role of doomscrolling in digital well-being and mental health.

## Figures and Tables

**Table 1:** Demographic and Behavioral Characteristics of the Sample (*n* = 793).

Characteristic	Category	*n*/M(SD)	%/Metric
Gender	Male	400	50.4
	Female	393	49.6
Age group	18–21 years	341	43.0
	22–25 years	452	57.0
Education	High school or less	140	17.7
	Undergraduate/Bachelor	555	70.0
	Postgraduate	98	12.4
Marital status	Single	611	77.0
	Married	159	20.1
	Divorced/Widowed	23	2.9
Primary platform	X/Twitter	107	13.5
	Snapchat	216	27.2
	Instagram	198	25.0
	TikTok	196	24.7
	YouTube	65	8.2
	Other	11	1.4
News-checking	Rarely	162	20.4
	Sometimes	208	26.2
	Often	264	33.3
	Very often	159	20.1
Composite scores	Doomscrolling M(SD)	4.36 (1.06)	7-point metric
	FoMO M(SD)	3.08 (0.78)	5-point metric
	BSMAS M(SD)	2.93 (0.75)	5-point metric
	PDWS total M(SD)	3.15 (0.72)	5-point metric

Note. Percentages are column percentages within the full sample. Composite scores are reported in their original response metrics. FoMO = fear of missing out; BSMAS = Bergen Social Media Addiction Scale; PDWS = perceived digital well-being scale.

**Table 2:** Factor-Analytic and Reliability Evidence for the Arabic Doomscrolling Scale.

Evidence Domain	Result
EFA suitability	KMO = 0.957; Bartlett χ^2^(105) = 2600.90, *p* < 0.001
Parallel analysis	One factor retained: first observed eigenvalue = 7.07 vs. 95th-percentile random eigenvalue = 1.41; second observed eigenvalue = 1.01 vs. random = 1.32
EFA one-factor solution	First factor accounted for 47.1% of total item variance; ML one-factor solution explained 43.5% of common variance; standardized loadings = 0.480–0.747
Primary ordinal CFA model (WLSMV)	scaled χ^2^(90) = 216.48, *p* < 0.001; CFI = 0.976; TLI = 0.972; RMSEA = 0.060, 90% CI [0.050, 0.070]; SRMR = 0.040; standardized loadings = 0.585–0.787
ML convergent CFA evidence	χ^2^(90) = 199.35, *p* < 0.001; χ^2^/df = 2.22; CFI = 0.959; TLI = 0.952; RMSEA = 0.055, 90% CI [0.045, 0.066]; SRMR = 0.081; standardized loadings = 0.574–0.767
Reliability and convergent-validity indicators	Cronbach’s α = 0.921 (full sample), α = 0.923 (CFA subsample), McDonald’s ω/CR = 0.924, AVE(WLSMV) = 0.476, AVE(ML) = 0.449

Note. EFA = exploratory factor analysis; CFA = confirmatory factor analysis; ML = maximum likelihood; KMO = Kaiser–Meyer–Olkin; CFI = comparative fit index; TLI = Tucker–Lewis index; RMSEA = root mean square error of approximation; SRMR = standardized root mean square residual; CR = composite reliability; AVE = average variance extracted; WLSMV = robust weighted least squares mean- and variance-adjusted estimator. ML results are reported as convergent evidence only.

**Table 3:** Ordinal Measurement Invariance of the Arabic Doomscrolling Scale Across Gender.

Model	Scaled χ^2^(df)	CFI	TLI	RMSEA	SRMR	ΔCFI	ΔRMSEA	ΔSRMR	Δχ^2^(df), *p*
Configural	326.38 (180)	0.974	0.970	0.064	0.049	—	—	—	—
Thresholds	383.75 (240)	0.975	0.978	0.055	0.049	+0.001	−0.009	0.000	37.32 (60), *p* = 0.99
Thresholds + loadings	361.53 (254)	0.981	0.984	0.046	0.050	+0.006	−0.009	+0.001	5.48 (14), *p* = 0.98
Strict (+residuals)	366.30 (269)	0.983	0.987	0.043	0.053	+0.002	−0.003	+0.003	8.63 (15), *p* = 0.90

Note. All models were estimated with WLSMV in the ordinal CFA framework using theta parameterization and the Wu and Estabrook (2016) identification sequence. χ^2^ values are scaled model test statistics. Δ values are changes relative to the preceding, less constrained model. Δχ^2^ values are scaled-and-shifted chi-square difference tests using the Satorra and Bentler (2010) correction. Negative ΔRMSEA values indicate improved approximate fit under added constraints. CFI = comparative fit index; TLI = Tucker–Lewis index; RMSEA = root mean square error of approximation; SRMR = standardized root mean square residual.

**Table 4:** Convergent, Criterion-Related, and Discriminant-Validity Evidence.

**Panel A. Associations of Doomscrolling with External Criteria**						
**Criterion measure**	**Coefficient**	**Estimate**	**95% CI**	***p***		
Fear of missing out	Pearson r	0.390	[0.329, 0.447]	<0.001		
Problematic social media use	Pearson r	0.521	[0.468, 0.570]	<0.001		
Perceived digital well-being	Pearson r	−0.376	[−0.435, −0.315]	<0.001		
Daily social media hours	Pearson r	0.262	[0.196, 0.325]	<0.001		
News-checking frequency	Spearman ρ	0.377	[0.312, 0.439]	<0.001		
**Panel B. Discriminant Validity Relative to Comparison Constructs**						
**Comparison**	**AVE DS**	**AVE comparison**	**Latent r**	**r^2^**	**HTMT**	**Fornell–Larcker**
Doomscrolling—Problematic SM use	0.441	0.337	0.624	0.389	0.627	Not satisfied
Doomscrolling—Fear of missing out	0.441	0.385	0.433	0.187	0.438	Satisfied
**Panel C. Additional Ordinal WLSMV Discriminant-Validity Test for Doomscrolling and Problematic Social Media Use in the Full Sample (*n* = 793)**						
**Model**	**scaled χ^2^(df)**	**CFI**	**RMSEA**	**SRMR**	**Model comparison**	
Two-factor model: DS and BSMAS freely correlated	397.91 (188)	0.983	0.038	0.031	Reference model	
Latent correlation fixed to 1.0	1139.46 (189)	0.925	0.080	0.059	Δχ^2^(1) = 78.04, *p* < 0.001	

Note. Panel A reports Pearson r for continuous criteria and Spearman ρ for the ordinal news-checking variable. Panel B uses estimates from the multi-construct ML measurement models; therefore, the AVE estimate for doomscrolling differs slightly from the single-factor CFA estimate in Table 2. Panel C reports an additional ordinal WLSMV nested-model comparison in the full sample (*n* = 793) for the key discriminant-validity test between doomscrolling and problematic social media use. In the freely correlated ordinal WLSMV two-factor model, the DS–BSMAS latent correlation was r = 0.626, 95% CI [0.571, 0.682]. DS = doomscrolling; BSMAS = Bergen Social Media Addiction Scale; AVE = average variance extracted; HTMT = heterotrait–monotrait ratio. The Fornell–Larcker criterion is satisfied when each construct’s square root of AVE exceeds the latent correlation.

**Table 5:** Standardized Associations from the Latent Incremental-Validity Model for Perceived Digital Well-Being.

Predictor	β	SE	z	*p*	95% CI
Doomscrolling	−0.269	0.057	−4.74	<0.001	[−0.380, −0.158]
Fear of missing out	−0.075	0.054	−1.40	0.163	[−0.180, 0.030]
Problematic social media use	−0.277	0.072	−3.86	<0.001	[−0.418, −0.136]
Daily social media hours	0.017	0.038	0.46	0.647	[−0.057, 0.092]
News-checking frequency	0.011	0.038	0.28	0.780	[−0.064, 0.085]
Gender (higher = female)	−0.033	0.037	−0.87	0.383	[−0.106, 0.041]
Age	0.042	0.038	1.11	0.268	[−0.033, 0.117]
Education	−0.031	0.038	−0.81	0.417	[−0.106, 0.044]

Note. β = standardized coefficient; SE = approximate standard error of the standardized coefficient; 95% CI = approximate 95% confidence interval. Standard errors and confidence intervals for standardized coefficients were obtained by scaling the standard errors returned for the unstandardized estimates and are therefore approximate. Doomscrolling, fear of missing out, and problematic social media use were modeled as latent variables; the remaining predictors were modeled as observed covariates. *n* = 793. The terms “association” and “incremental validity” are used because the design is cross-sectional and does not support causal inference. Gender was coded as 1 = male and 2 = female; education level was coded as 1 = high school or less, 2 = undergraduate/bachelor, and 3 = postgraduate; and news-checking frequency was coded as 1 = rarely, 2 = sometimes, 3 = often, and 4 = very often.

## Data Availability

The item-level data supporting the findings of this study are not publicly available because the ethics approval and informed-consent conditions do not allow unrestricted public deposition of participant-level survey responses. The dataset contains self-report responses from Saudi young-adult participants on digital behavior and perceived well-being; therefore, public sharing is restricted to protect participant confidentiality and comply with the approved consent conditions. De-identified item-level data may be made available from the corresponding author upon reasonable academic request, subject to institutional ethical approval and, where applicable, a data-sharing agreement. Analysis syntax used to conduct the reported psychometric analyses may also be made available from the corresponding author upon reasonable request.

## Supplementary Materials

The following supporting information can be downloaded at https://www.mdpi.com/article/10.3390/bs16091528/s1. Table S1: Item-Level Descriptive Statistics for the Arabic Doomscrolling Scale; Table S2: Standardized Factor Loadings for the Arabic Doomscrolling Scale; Table S3: Equivalence Tests for the EFA and CFA Subsamples; Table S4: Psychometric Adequacy of the External Measures Used for Validity Analyses; Table S5: Graded Response Model Discrimination and Gender Differential Item Functioning; Section S1: Arabic and English Doomscrolling Scale Items Used in the Present Study; and Section S2: English and Arabic Blank Informed Consent Form.

## References

[B1-behavsci-16-01528] Al-Menayes J. (2016). The Fear of Missing out Scale: Validation of the Arabic version and correlation with social media addiction. International Journal of Applied Psychology.

[B2-behavsci-16-01528] American Educational Research Association, American Psychological Association, National Council on Measurement in Education (2014). Standards for educational and psychological testing.

[B3-behavsci-16-01528] Andreassen C. S., Billieux J., Griffiths M. D., Kuss D. J., Demetrovics Z., Mazzoni E., Pallesen S. (2016). The relationship between addictive use of social media and video games and symptoms of psychiatric disorders: A large-scale cross-sectional study. Psychology of Addictive Behaviors.

[B4-behavsci-16-01528] Boateng G. O., Neilands T. B., Frongillo E. A., Melgar-Quiñonez H. R., Young S. L. (2018). Best practices for developing and validating scales for health, social, and behavioral research: A primer. Frontiers in Public Health.

[B5-behavsci-16-01528] Capraro V., Globig L., Rausch Z., Rathje S., Wormley A. S., Olson J., Ross R. M., Aşçı S., Bouguettaya A., Burnell K., Choukas-Bradley S., Fardouly J., Kowert R., Lopez R. B., Maheux A. J., Mirea D.-M., Ozimek P., Selterman D. F., Thiagarajan T., Van Bavel J. J. (2025). A collective review on some potential negative impacts of smartphone and social media use on adolescent mental health: Results from a Delphi process. PsyArXiv.

[B6-behavsci-16-01528] Chen F. F. (2007). Sensitivity of goodness of fit indexes to lack of measurement invariance. Structural Equation Modeling: A Multidisciplinary Journal.

[B7-behavsci-16-01528] Cheung G. W., Rensvold R. B. (2002). Evaluating goodness-of-fit indexes for testing measurement invariance. Structural Equation Modeling: A Multidisciplinary Journal.

[B8-behavsci-16-01528] Communications, Space and Technology Commission (2025). CST issued the Saudi internet report 2024.

[B9-behavsci-16-01528] El Abiddine F. Z., Aljaberi M. A., Alduais A., Lin C.-Y., Vally Z., Griffiths M. D. (2025). The psychometric properties of the Arabic Bergen Social Media Addiction Scale. International Journal of Mental Health and Addiction.

[B10-behavsci-16-01528] Elsokkary E. M. (2026). Psychometric properties of the Arabic Perceived Digital Well-Being Scale (PDWS) in Saudi young adults. PLoS ONE.

[B11-behavsci-16-01528] Fornell C., Larcker D. F. (1981). Evaluating structural equation models with unobservable variables and measurement error. Journal of Marketing Research.

[B12-behavsci-16-01528] Güme S. (2024). Doomscrolling: A review. Psikiyatride Güncel Yaklaşımlar-Current Approaches in Psychiatry.

[B13-behavsci-16-01528] International Test Commission (2017). The ITC guidelines for translating and adapting tests (Second edition). International Journal of Testing.

[B14-behavsci-16-01528] Kaya B., Griffiths M. D. (2025). Intolerance of uncertainty and mental wellbeing: The mediating and moderating role of doomscrolling. Behaviour & Information Technology.

[B15-behavsci-16-01528] Kemp S. (2025). Digital 2025: Saudi Arabia. *DataReportal*.

[B16-behavsci-16-01528] Kushlev K., Leitao M. R. (2020). The effects of smartphones on well-being: Theoretical integration and research agenda. Current Opinion in Psychology.

[B17-behavsci-16-01528] Neijzen M. (2024). The epistemic value of doombehaviour: Beyond the prudential consequences of doomscrolling, doomchecking, and doomsurfing. Synthese.

[B18-behavsci-16-01528] Odgers C. L., Jensen M. R. (2020). Annual research review: Adolescent mental health in the digital age: Facts, fears, and future directions. Journal of Child Psychology and Psychiatry.

[B19-behavsci-16-01528] Orben A., Przybylski A. K. (2019). The association between adolescent well-being and digital technology use. Nature Human Behaviour.

[B20-behavsci-16-01528] Przybylski A. K., Murayama K., DeHaan C. R., Gladwell V. (2013). Motivational, emotional, and behavioral correlates of fear of missing out. Computers in Human Behavior.

[B21-behavsci-16-01528] Putnick D. L., Bornstein M. H. (2016). Measurement invariance conventions and reporting: The state of the art and future directions for psychological research. Developmental Review.

[B22-behavsci-16-01528] Rosič J., Carbone L., Vanden Abeele M. M. P., Lobe B., Vandenbosch L. (2024). Measuring digital well-being in everyday life among Slovenian adolescents: The Perceived Digital Well-Being in Adolescence Scale. Journal of Children and Media.

[B23-behavsci-16-01528] Satici S. A., Gocet Tekin E., Deniz M. E., Satici B. (2023). Doomscrolling Scale: Its association with personality traits, psychological distress, social media use, and wellbeing. Applied Research in Quality of Life.

[B24-behavsci-16-01528] Satorra A., Bentler P. M. (2010). Ensuring positiveness of the scaled difference chi-square test statistic. Psychometrika.

[B25-behavsci-16-01528] Sharma B., Lee S. S., Johnson B. K. (2022). The dark at the end of the tunnel: Doomscrolling on social media newsfeeds. Technology, Mind, and Behavior.

[B26-behavsci-16-01528] Soraci P., Griffiths M. D., Bevan N., Pisanti R., Trovato M., Servidio R., D’Aleo E., Campedelli L., Gallo F., Satici S. A. (2025). Psychometric analysis of the Italian Doomscrolling Scale: Associations with problematic social media use, psychological distress, and mental well-being. Current Psychology.

[B27-behavsci-16-01528] Vanden Abeele M. M. P. (2021). Digital wellbeing as a dynamic construct. Communication Theory.

[B28-behavsci-16-01528] Vera Cruz G., Liberacka-Dwojak M., Wiłkość-Dębczyńska M., Aktaş Terzioğlu M., Farchione T., Lecomte T., Ingram S., Khan R., Khazaal Y. (2025). Perceived Digital Well-Being Scale in the United States and United Kingdom: Psychometric validation study. JMIR Mental Health.

[B29-behavsci-16-01528] Wu H., Estabrook R. (2016). Identification of confirmatory factor analysis models of different levels of invariance for ordered categorical outcomes. Psychometrika.

