# Supplementary material for: Psychometric Validation of the Arabic Doomscrolling Scale Among Saudi Young-Adult Social Media Users: Factor Structure, Measurement Invariance Across Gender, and Digital Correlates
Source: Behav Sci (Basel). 2026 Aug 30;16(9):1528. doi: 10.3390/bs16091528 (PMC13603113; doi:10.3390/bs16091528)
Supplement: Supplementary file 1 [file behavsci-16-01528-s001.zip › behavsci-4427816-supplementary.pdf]

Supplementary Materials

Psychometric Validation of the Arabic Doomscrolling Scale Among Saudi Young-Adult Social Media Users: Factor Structure, Measurement Invariance Across Gender, and Digital Correlates

Emadeldin M. Elsokkary

This supplementary file contains the supplementary tables referenced in the manuscript. Table numbering follows the manuscript text.

Supplementary Table S1. Item-Level Descriptive Statistics for the Arabic Doomscrolling Scale

Item	M	SD	Skewness	Kurtosis	r_it	α if deleted
DS1	4.41	1.45	−0.26	−0.21	.70	.914
DS2	4.28	1.65	−0.22	−0.69	.66	.915
DS3	4.53	1.43	−0.29	−0.37	.65	.915
DS4	4.34	1.55	−0.19	−0.56	.68	.914
DS5	4.44	1.59	−0.29	−0.62	.60	.917
DS6	4.50	1.48	−0.16	−0.49	.67	.914
DS7	4.19	1.57	−0.14	−0.72	.71	.913
DS8	4.35	1.57	−0.16	−0.47	.61	.916
DS9	4.08	1.62	−0.12	−0.74	.60	.916
DS10	4.44	1.57	−0.19	−0.64	.72	.913
DS11	4.20	1.61	−0.17	−0.62	.56	.918
DS12	4.42	1.62	−0.29	−0.78	.61	.916
DS13	4.57	1.51	−0.21	−0.75	.58	.917
DS14	4.27	1.42	−0.15	−0.36	.51	.919
DS15	4.40	1.45	−0.24	−0.10	.65	.915

Note. M = mean; SD = standard deviation; r_it = corrected item–total correlation; α if deleted = Cronbach's alpha with the item removed. Items are labeled DS1–DS15 to preserve correspondence with the source instrument.

Supplementary Table S2. Standardized Factor Loadings for the Arabic Doomscrolling Scale

Item	EFA loading	CFA loading (WLSMV)	CFA loading (ML)
DS1	.684	.786	.767
DS2	.711	.674	.661
DS3	.657	.741	.711
DS4	.730	.715	.693
DS5	.579	.670	.657
DS6	.747	.712	.676
DS7	.745	.760	.735
DS8	.605	.681	.664
DS9	.586	.686	.663
DS10	.740	.787	.766
DS11	.584	.586	.574
DS12	.614	.664	.649
DS13	.650	.604	.581
DS14	.480	.585	.578
DS15	.711	.658	.641

Note. EFA = exploratory factor analysis (maximum likelihood extraction, EFA subsample); CFA (WLSMV) = ordinal confirmatory factor analysis using the robust weighted least squares mean- and variance-adjusted estimator in the CFA subsample; CFA (ML) = maximum likelihood confirmatory factor analysis reported as convergent evidence. All CFA loadings were statistically significant at $p < .001$.

Supplementary Table S3. Equivalence Tests for the EFA and CFA Subsamples

Variable	Test	Statistic	p
Gender	$\chi^2(1)$	0.36	.546
Age group	$\chi^2(1)$	0.21	.645
Education	$\chi^2(2)$	0.54	.763
Age	t(791)	−0.35	.724
Daily social media hours	t(791)	1.43	.155
News-checking frequency	Mann–Whitney U test	Not reported	.204

Note. The sample was randomly partitioned into an EFA subsample (n = 396) and a CFA subsample (n = 397) using a fixed seed (12345). The nonsignificant results support comparability of the two subsamples for cross-validation.

Supplementary Table S4. Psychometric Adequacy of the External Measures Used for Validity Analyses

Measure	Factor structure / analytic basis	Standardized loading range	Cronbach's α	CR / ω	AVE	Use in the current study
Fear of missing out (FoMO)	Single factor (parallel analysis retained one factor)	.533–.689	.860	.861	.385	Convergent criterion; latent predictor in the incremental-validity model
Bergen Social Media Addiction Scale (BSMAS)	Single factor; modeled as a latent factor in the multi-construct discriminant-validity and incremental-validity ML models	.475–.685	.744	.750	.337	Discriminant comparison; latent predictor in the incremental-validity model
Perceived digital well-being (PDWS, total)	Total scale; modeled as a latent factor with the three domain scores as indicators in the incremental-validity model	.674–.709	.887	.729	.473	Latent outcome (criterion)
Emotional domain	Domain composite (indicator)	—	.860	—	—	Indicator of the PDWS latent factor
Social domain	Domain composite (indicator)	—	.801	—	—	Indicator of the PDWS latent factor
Cognitive domain	Domain composite (indicator)	—	.764	—	—	Indicator of the PDWS latent factor

Note. CR = composite reliability; ω = McDonald's omega; AVE = average variance extracted; dashes indicate indices not estimated for that row. AVE, HTMT, and latent correlations reported in the discriminant-validity analyses were derived from the multi-construct maximum likelihood (ML) measurement models; loading ranges and AVE for the external measures are reported here for transparency. For FoMO and BSMAS, standardized loading ranges are from one-factor ML measurement models. For the PDWS latent factor, the loading range, CR/ω, and AVE are based on the three domain-score indicators used in the incremental-validity model; the domain rows report internal-consistency reliabilities of the item composites that form those indicators. N = 793.

Supplementary Table S5. Graded Response Model Discrimination and Gender Differential Item Functioning

Item	Discrimination (a)	ΔR² (DIF)	DIF classification
DS1	1.94	.0007	Negligible
DS2	1.67	.0003	Negligible
DS3	1.70	.0005	Negligible
DS4	1.82	.0016	Negligible
DS5	1.38	.0007	Negligible
DS6	1.79	.0019	Negligible
DS7	2.09	.0000	Negligible
DS8	1.46	.0006	Negligible
DS9	1.47	.0001	Negligible
DS10	2.09	.0006	Negligible
DS11	1.23	.0010	Negligible
DS12	1.46	.0005	Negligible
DS13	1.40	.0004	Negligible
DS14	1.13	.0006	Negligible
DS15	1.69	.0007	Negligible

Note. a = GRM item discrimination parameter; ΔR^2 = change in R^2 attributable to gender (uniform plus nonuniform DIF) from ordinal logistic regression. "Negligible" follows the Jodoin–Gierl classification. No item was statistically significant after Bonferroni correction. GRM = graded response model; DIF = differential item functioning.

Section S1. Doomscrolling Scale Items Used in the Present Study

English Version

Authors: Sharma, Lee, and Johnson

Year: 2022

Instruction: Please indicate the extent to which you agree or disagree with each statement regarding your experiences with negative news on social media.

Response options: 1 = Strongly disagree; 2 = Disagree; 3 = Somewhat disagree; 4 = Neither agree nor disagree; 5 = Somewhat agree; 6 = Agree; 7 = Strongly agree.

No.	Item	Response options
1	I feel an urge to seek bad news on social media, more and more often.	1 2 3 4 5 6 7
2	I lose track of time when I read bad news on social media.	1 2 3 4 5 6 7
3	I constantly refresh my newsfeeds to see if something bad happened.	1 2 3 4 5 6 7
4	I stay up late at night trying to find more negative news.	1 2 3 4 5 6 7
5	Reading negative news on social media is more of a habit now.	1 2 3 4 5 6 7
6	When I am online, I feel tense as if something bad is going to happen soon.	1 2 3 4 5 6 7
7	I constantly feel panicked while scrolling on my device.	1 2 3 4 5 6 7
8	I unconsciously check my newsfeeds for bad news.	1 2 3 4 5 6 7
9	Even if my newsfeed says I am all caught up, I just keep scrolling for negative news.	1 2 3 4 5 6 7
10	I find myself continuously browsing negative news.	1 2 3 4 5 6 7
11	I check social media in the morning to see what bad things have happened.	1 2 3 4 5 6 7
12	I feel like I am addicted to negative news.	1 2 3 4 5 6 7
13	My social media searches probably make my newsfeeds more negative.	1 2 3 4 5 6 7
14	I am terrified by what I see on social media but I cannot look away.	1 2 3 4 5 6 7
15	It is difficult to stop reading negative news on social media.	1 2 3 4 5 6 7

النسخة العربية

مقياس التصفح القهري للأخبار السلبية

معدو المقياس: شارما ولي وجونسون

السنة: 2022م

التعليمات: يرجى تحديد درجة موافقتك أو عدم موافقتك على كل عبارة من العبارات الآتية، وذلك في ضوء خبرتك في متا□عة الأخبار السلبية على وسائل التواصل الاجتماعي.

□دائل الاستجا□ة: 1 = لا أوافق□شدة؛ 2 = لا أوافق؛ 3 = لا أوافق إلى□د ما؛ 4 = لا أوافق ولا أعارض؛ 5 = أوافق إلى□د ما؛ 6 = أوافق؛ 7 = أوافق□شدة.

م	العبارة	بدائل الاستجابة
1	أشعر□رغبة متزايدة في البحث عن الأخبار السيئة على وسائل التواصل الاجتماعي.	1 2 3 4 5 6 7
2	أفقد الإ□ساس□الوقت عندما أقرأ الأخبار السيئة على وسائل التواصل الاجتماعي.	1 2 3 4 5 6 7
3	أقوم□تحديث موجزات الأخبار□استمرار لأرى ما إذا كان قد□دث شيء سيئ.	1 2 3 4 5 6 7
4	أسهر □تى وقت متأخر من الليل محاولًا العثور على مزيد من الأخبار السلبية.	1 2 3 4 5 6 7
5	أصبحت قراءة الأخبار السلبية على وسائل التواصل الاجتماعي عادة لديّ.	1 2 3 4 5 6 7
6	عندما أكون متصلًا□الإنترنت، أشعر□التوتر كما لو أن شيئًا سيئًا سيحدث قريبًا.	1 2 3 4 5 6 7
7	أشعر□الذعر□استمرار أثناء التصفح على جهازي.	1 2 3 4 5 6 7
8	أتفقد موجزات الأخبار دون وعي□حثًا عن الأخبار السيئة.	1 2 3 4 5 6 7
9	□تى إذا أظهر موجز الأخبار أنني اطلعت على كل المستجدات، أستمر في التصفح□حثًا عن الأخبار السلبية.	1 2 3 4 5 6 7
10	أجد نفسي أتصفح الأخبار السلبية□استمرار.	1 2 3 4 5 6 7
11	أتفقد وسائل التواصل الاجتماعي في الصباح لأعرف ما الأشياء السيئة التي □دثت.	1 2 3 4 5 6 7
12	أشعر وكأنني مدمن على الأخبار السلبية.	1 2 3 4 5 6 7
13	من المحتمل أن تجعل عمليات□حثي على وسائل التواصل الاجتماعي موجزات الأخبار لديّ أكثر سلبية.	1 2 3 4 5 6 7
14	أشعر□الرعب مما أراه على وسائل التواصل الاجتماعي، لكنني لا أستطيع التوقف عن المتا□عة.	1 2 3 4 5 6 7
15	يصعب عليّ التوقف عن قراءة الأخبار السلبية على وسائل التواصل الاجتماعي.	1 2 3 4 5 6 7

Section S2. Blank Informed Consent Form

(English Version)

Study Title: Psychometric Validation of the Arabic Doomscrolling Scale Among Saudi Young Adults: Factor Structure, Measurement Invariance Across Gender, and Digital Correlates

This is a blank copy of the electronic informed consent statement presented before the online survey.

Principal Investigator

Emadeldin M. Elsokkary, Department of Psychology, College of Social Sciences, Imam Mohammad Ibn Saud Islamic University (IMSIU), Riyadh, Saudi Arabia. Email: emelsokkary@imamu.edu.sa

Purpose of the Study

You are invited to participate in a research study examining doomscrolling, fear of missing out, problematic social media use, and perceived digital well-being among Saudi young adults. The study aims to validate an Arabic measure of doomscrolling and to understand how habitual engagement with negative online news relates to other patterns of social media use and to perceived digital well-being.

Procedures

If you agree to participate, you will complete an anonymous online questionnaire. The questionnaire includes demographic and social media use questions and self-report scales related to doomscrolling, fear of missing out, problematic social media use, and perceived digital well-being. Completion is expected to take approximately 10–15 minutes.

Voluntary Participation and Withdrawal

Participation is entirely voluntary. You may choose not to participate or may stop completing the survey before submission without penalty. Because responses are collected anonymously, once the survey is submitted it may not be possible to identify and remove a specific individual response.

Risks and Benefits

The study involves minimal risk. Some questions ask about ordinary experiences of smartphone and social media use, including engagement with negative or distressing online news. If you feel uncomfortable, you may stop completing the survey at any time before submission. There is no direct personal benefit, but your participation may contribute to research on digital behavior and digital well-being among young adults.

Confidentiality and Data Protection

No personally identifying information will be requested. Responses will be collected anonymously and reported only in aggregate form for research purposes. Data will be used for scholarly analysis and publication, and access will be limited to the researcher.

Ethical Approval

This study was approved by the Institutional Review Board of Imam Mohammad Ibn Saud Islamic University (IMSIU) (Registration No. HAPO-01-R-061; protocol/project code: 405/2025; approval date: 20 November 2025).

Consent Statement

By selecting "I agree" and proceeding to the survey, you confirm that you have read the information above, are a Saudi national, are between 18 and 25 years of age, use social media platforms, understand that participation is voluntary, and agree to participate in this study.

☐ I agree to participate in this study.

☐ I do not agree to participate in this study.

نموذج الموافقة المستنيرة الفارغ

(Arabic Version)

عنوان الدراسة: التحقق السيكومتري من النسخة العربية لمقياس التمرير الكارثي (Doomscrolling) لدى الشباب السعوديين: البنية العاملية، وثبات القياس، والمرتبطات الرقمية

هذه نسخة فارغة من نص الموافقة المستنيرة الإلكترونية الذي عُرض قبل بدء الاستبيان الإلكتروني.

الباحث

عماد الدين محمد السكري، قسم علم النفس، كلية العلوم الاجتماعية، جامعة الإمام محمد□ن سعود الإسلامية، الرياض، المملكة العر□ية السعودية. البريد الإلكتروني: emelsokkary@imamu.edu.sa

هدف الدراسة

ندعوك للمشاركة في دراسة□حثية تتناو□ التمرير الكارثي (Doomscrolling)، والخوف من فوات الشيء، والاستخدام الإشكالي لوسائل التواصل الاجتماعي، والرفاه الرقمي المُدرَك لدى الشبا□ السعوديين. وتهدف الدراسة إلى التحقق من الخصائص السيكومترية لمقياس عر□ي للتمرير الكارثي، وإلى فهم كيفية ارتباط الانهماك المعتاد في متا□عة الأخبار السلبية على الإنترنت□أنماط أخرى لاستخدام وسائل التواصل الاجتماعي و□الرفاه الرقمي المُدرَك.

إجراءات المشاركة

إذا وافقت على المشاركة، فستُكمِل استبيانًا إلكترونيًا مجهو□ الهوية. ويتضمن الاستبيان أسئلة ديموغرافية وأسئلة عن استخدام وسائل التواصل الاجتماعي، ومقاييس تقرير ذاتي تتعلق□التمرير الكارثي، والخوف من فوات الشيء، والاستخدام الإشكالي لوسائل التواصل الاجتماعي، والرفاه الرقمي المُدرَك. ومن المتوقع أن تستغرق الإجا□ة □والي 10–15 دقيقة.

طوعية المشاركة والانسحاب

المشاركة في هذه الدراسة طوعية تمامًا. ويمكنك عدم المشاركة أو التوقف عن إكما□ الاستبيان قبل إرساله دون أي عقو□ة. ونظرًا لأن الاستجا□ات تُجمع□صورة مجهولة، فقد لا يكون من الممكن تحديد استجا□ة فردية معينة و□ذفها□عد إرسا□ الاستبيان.

المخاطر والفوائد

لا تنطوي الدراسة على مخاطر تتجاوز الحد الأدنى. وقد تتناو□□عض الأسئلة خبرات عادية مرتبطة□استخدام الهاتف الذكي ووسائل التواصل الاجتماعي،□ما في ذلك متا□عة الأخبار السلبية أو المزعجة على الإنترنت. وإذا شعرت□عدم الارتياح، فيمكنك التوقف عن إكما□ الاستبيان في أي وقت قبل إرساله. ولا توجد فائدة شخصية مباشرة، إلا أن مشاركتك قد تسهم في تطوير المعرفة البحثية□و□ السلوك الرقمي والرفاه الرقمي لدى الشبا□.

السرية وحماية البيانات

لن يُطلب منك تقديم أي معلومات تحدد هويتك الشخصية. ستُجمع الاستجا□ات□صورة مجهولة، وستُعرض النتائج في صورة إجمالية فقط لأغراض البحث العلمي. وستُستخدم البيانات للتحليل والنشر العلمي، وسيكون الوصو□ إليها محدودًا□البا□ث.

الموافقة الأخلاقية

□صلت الدراسة على موافقة لجنة أخلاقيات البحث في جامعة الإمام محمد□ن سعود الإسلامية (رقم التسجيل: HAPO-01-R-061؛ رمز/رقم المشروع: 2025/405؛ تاريخ الموافقة: 20 نوفمبر 2025).

عبارة الموافقة

□اختيارك عبارة «أوافق» والمتا□عة إلى الاستبيان، فإنك تؤكد أنك قرأت المعلومات أعلاه، وأنك سعودي/سعودية، وأن عمرك□ين 18 و25 سنة، وأنك تستخدم وسائل التواصل الاجتماعي، وأنك تفهم أن المشاركة طوعية، وأنك توافق على المشاركة في هذه الدراسة.

☐ أوافق على المشاركة في هذه الدراسة.

☐ لا أوافق على المشاركة في هذه الدراسة.
